# Clinical Studies with Non-Nauseating Doses of Ipecacuanha, Chiefly in Intermittents

**Published:** 1875-07

**Authors:** Alfred A. Woodhull

**Affiliations:** Assistant Surgeon U. S. Army


### CLINICAL STUDIES WITH NON-NAUSEATING DOSES OF IPECACUANHA, CHIEFLY IN INTERMITTENTS.

(Concluded, from page 146 June number.)

BY ALFRED A. WOODHULL, M.D., ASSISTANT SURGEON U. S. ARMY.

*An Abstract from a Special Report to the Surgeon General U. S. Army, read before the Atlanta Academy of Medicine.*

CASE VI.—J. C. A fortnight after reaching Alabama, in September, this man was taken with bilious fever, with which he was sick three weeks. He says he was languid and indisposed during the remainder of his absence, but, returning late in November, felt well until 15th December, 1874, when he had a light chill at 10 A.M., with fever lasting until 3 P.M. He had no chill on 16th.

17th December. Had a severer chill with less fever. Temp. 2 P.M., 102 4-5. Admitted hospital and given one grain of ipecacuanha every three hours.

18th.—Temp. A.M., 97 1-5; P.M., 98 3-5. ℞. Ipecac. gr. v., opii gr. $\frac{1}{4}$ at noon.

19th.—Temp. A.M., 98; 1 P.M., 99 1-5; P.M., 101 4-5. A slight rigor but not a marked chill to-day. ℞. Ipecac. gr. ij every two hours.

20th.—Temp. 7 A.M., 97 4-5; 1 P.M., 97 4-5; 6 P.M., 98 2-5. Treatment continued.

21st.—Temp. 7 A.M., 97 4-5; 12 M., 96 4-5; 6 P.M., 98 3-5. No disagreeable sensation. ℞. Ipecac. gr. x., opii gr. ½, 10 A.M.; deferred dinner until 2 P.M.; ipecac. gr. v., opii gr. ¼, 8:30 P.M.

22d.—Temp. 7 A.M., 98. Duty. Directed to take a two-grain pill every three hours during the day for three weeks. The disease has not recurred.

CASE VII.—E. P. While on detached service in Alabama this man was ill three weeks with bilious fever, from which he was only convalescent when he returned to this post late in November.

27th December, 1874.—Reports sick. Says he had a light chill while on guard 24th, and had a severe one on 26th. Has taken no medicine of any kind. Admitted hospital and given two grains of ipecacuanha every two hours. Temp. A.M., 98 3-5; P.M., 99 2-5.

28th.—Temp. A.M., 98 3-5; 1 P.M., 99 2-5; P.M., 100 1-5. Had no chill but there was some fever between ten and one o'clock. Took two grains every two hours and five grains with one-fourth grain opium at night.

29th.—Temp. A.M., 98 2-5; P.M., 98 3-5. Two grains bi-hourly.

30th.—Temp. A.M., 97 2-5; P.M., 99. Slight chilly feeling. Treatment continued.

31st.—Temp. A.M., 97 3-5; P.M., 97 4-5. Continue treatment.

1st January, 1875.—Temp. A.M., 98 1-5; P.M., 97 4-5. 2d, A.M., 97 2-5; P.M., 98 4-5. 3d, A.M., 97 3-5; P.M., 99. 4th, A.M., 98. Returned to quarters. He took two grains every two hours each day without inconvenience. No chilly sensations occurred after 30th ult., and he was returned to duty 6th January with instructions to continue using the medicine for three weeks.

30th March, 5 P.M.—This man reports as just passing out of a chill. Temperature 101 3-5. ℞. Ipecac. gr. j every three hours.

31st.—Temp. 7 A.M., 98; 11 A.M., 98 3-5; 3 P.M., 97 4-5; 7 P.M., 98 3-5. No chill. Treatment continued.

1st April.—Temp. 7 A.M., 98 3-5; 10 A.M., 98 3-5; 12 M., 98 2-5; 2 P.M., 98 1-5; 4 P.M., 98; 7 P.M., 99 2-5. No chill. Slightly nauseated and pills reduced to one every four hours.

2d.—Temp. 7 A.M., 98; 2 P.M., 98 2-5; 7 P.M., 98 3-5. Treatment continued.

3d.—Temp. 7 A.M., 97 3-5; 10 A.M., 98; 2 P.M., 98 1-5; 6 P.M., 101 1-5. Notwithstanding this rise there was no abnormal sensation. Treatment continued.

4th.—Temp. 7 A.M., 97 4-5. 5th, 7 A.M., 98 1-5; P.M., 98 1-5. Returned to quarters.

6th.—Temp. A.M., 96 3-5; P.M., 99. Notwithstanding this irregularity there was no feeling of illness on the man's part, and he was returned to duty, 9th April, with instructions to continue using the pills.

CASE VIII.—W. M. This man was sick in Alabama about four weeks. After his return, 28th November, 1874, he felt pretty well until about 10th December, when he became feverish and had malarial symptoms daily, although remaining on duty until 29th. He then reported sick, complaining of the train of symptoms which had culminated in all the indications of an attack of ague. ℞. Pulv. rhei., pulv. ipecac., hydg. chl. mit. aa. gr. v. The medicine both vomited and purged, and he then felt better. Temp. A.M., 99; P.M., 99 2-5.

30th.—Admitted hospital and given two grains ipecacuanha bi-hourly, and at night five grains with one-quarter grain opium. Temp. A.M., 98 3-5; P.M., 100 1-5.

31st.—Temp. A.M., 98 1-5; P.M., 99. Two grains bi-hourly.

1st January, 1875.—Temp. A.M., 98; P.M., 98 3-5. 2d, A.M., 97 4-5; P.M., 98 3-5. 3d, A.M., 97 4-5. Treatment continued as last noted. Has had no chill nor has the medicine nauseated. Returned to duty and to take one grain every two hours for several weeks. The aguish character of this case is not well marked in the foregoing record, but the man's history and appearance presented unequivocal evidence of malarial contamination.

16th January.—Says he took four or five pills daily until today, when he had a fever.

17th.—Temp. A.M., 97. To take ipecac. gr. v., opii gr. $\frac{1}{4}$ at 9 A.M., 4 and 7 P.M. and 6 A.M. to-morrow.

18th.—Attempted to go on with his work, but became too ill and was excused 10 A.M. Had no distinct chill, but his temperature at that hour was 104 1-5; 2 P.M., 101 3-5; 7 P.M., 101 1-5. ℞. Ipecac. gr. x., opii gr. ss. at 8:30 P.M.

19th.—℞. Ipecac. gr. v., opii gr. $\frac{1}{4}$ at 6 A.M., 11 A.M. and 3 P.M. Temp. A.M., 98 1-5; P.M., 100 4-5. Had a slight fever but no chill. Under a misapprehension took a five-and-a-quarter grain pill at 8 and another at 8:30 P.M., soon after which he vomited about a pint of greenish fluid. Fell asleep immediately afterward and slept all night.

20th.—Temperature A.M., 102 1-5, notwithstanding which he felt well. 9 A.M., 102 2-5; 1 P.M., 100 3-5; 6 P.M., 99 3-5. He took ipecac. gr. v., opii gr. $\frac{1}{4}$ at each of the hours just specified.

21st.—A.M., 98; P.M., 103 4-5. Notwithstanding this elevation of temperature he was entirely unconscious of more than a slight increase, and insisted that he felt quite well. Took the $5\frac{1}{4}$ gr. pill at 9 A.M., 1 and 6 P.M.

22d.—Temp. A.M., 98 3-5; 1 P.M., 98. Took $10\frac{1}{2}$ grs. 6 A.M., $5\frac{1}{4}$ 1 and 6 P.M. 23d. Returned to duty.

CASE IX.—Mrs. ......, of good general health, was confined on the morning of 6th January, 1875, having a natural and easy labor. The after-pains were somewhat more sharp and constant than usual, but nothing especial occurred until 10th, when she experienced a slight rigor followed by a perceptible fever and by wandering but severe general pains in all the limbs. There was a little subsequent perspiration. It was not convenient to use the thermometer, but her pulse during the exacerbation reached 109. This lady, on several previous occasions, when her general strength had become temporarily impaired, had mild attacks of intermittent fever; and I looked upon this as similar, and not as milk fever, as it may occur to the reader. In view of Trousseau's praises of this drug in the puerperal state, as well as its presumed action in intermittents, she was ordered ipecac. gr. xx., opii gr. j, in pil. iv.; one to be taken at 8 P.M., 1 A.M. and 6 A.M.

11th.—Slept well and was not materially nauseated. The fourth pill was taken at 10 A.M., after which two grains of ipecacuanha was ordered every two hours. While taking the larger doses she was cautioned about drinking; but, not understanding that the same rule applied to the smaller pills, she took one immediately before tea, and that meal was, consequently, rejected There was no attendant nor subsequent nausea. During the night she took four doses.

12th.—Ipecac. gr. ij every four hours during the day, and in the night at 10 P.M., 3 and 6 A.M.

13th.—The medicine was repeated at 10:30 A.M., 3:30 and 9 P.M. and 6 A.M.

14th.—No recurrence of the paroxysm happening, the medicine was suspended.

During its exhibition the only emesis was that noted, and although occasional nausea occurred, it was not severe. The patient made a speedy and uniform convalescence.

CASE X.—C. L. On sick report since 11th January, 1875, with a slight gastric and intestinal derangement, complained, on 15th, of a chilly sensation. Temperature A.M., 97 2-5; P.M., 100 3-5. ℞. Ipecac. gr. v., opii gr. ¼, at 8 P.M.

16th.—Temp. A.M., 98 2-5; P.M., 99 1-5. To take the same dose at 6 and 10 A.M. and 5 P.M. Feels well to-day.

17th.—Temp. A.M., 98 1-5; P.M., 98 3-5. Repeat pill at 6 and 10 A.M. and 8:30 P.M.

18th.—Temp. A.M., 98; P.M., 99 1-5. Pill repeated at 6 A.M. and 8:30 P.M.

19th.—Temp. A.M., 96 3-5; 11 A.M., 99. Notwithstanding these variations of temperature, he feels well and has felt thus for several days. Returned to duty and directed to take five grains of ipecacuanha, without opium, night and morning, for some time.

CASE XI.—J. L. This man, now thirty-five years of age and of fifteen years' service, although of good habits and a faithful and excellent soldier, has had his constitution and general health weakened by the campaigns and privations through which he has passed. He suffered with a severe remittent attack in Alabama, and was on the sick report here 13–16 December, 1874, with a quotidian intermittent, for which he was treated by Asst. Surg. Maus with a combination of quinine and ipecacuanha.

14th January, 1875.—Reports having a violent chill yesterday. (Being married he is treated in quarters.) Temp. A.M., 97 3-5; P.M., 98 3-5. Took ipecac. gr. x., opii gr. ss. at 11 A.M. and 1 P.M. There was neither nausea nor vomiting.

15th.—Temp. A.M., 97 3-5; 4 P.M., 103 2-5; 6 P.M., 101 2-5. A severe chill, but lighter than the preceding, occurred about noon. Took ipecac. gr. ij. every two hours, and ipecac. gr. v., opii gr. ¼, at 8:30 P.M.

16th.—Temp. A.M., 96 4-5; M., 97 4-5; P.M., 98. No chill. Took one pill of ipecac. gr. v., opii gr. ¼, at 6 A.M., 10 A.M., 4 P.M. and two at 8:30 P.M.

17th —Temp. A.M., 97 2-5; M., 98 1-5; P.M., 98. Took the same pill at 6 A.M., two at 11 A.M., and one at 1 and 7 P.M. There was no vomiting and no chill, but a profuse cold sweat occurred in the night.

18th.—Temp. A.M., 98; P.M., 98 3-5. The usual pill at 9 A.M. and 3 and 8:30 P.M. Had no chill nor fever, nor any perspiration at night.

19th.—Temp. A.M., 98 4-5; P.M, 99 4-5. Took the same pill at 11 A.M. and 3 and 7 P.M. His head aching and his bowels being constipated, took pil. cath. c. no. iij at bedtime. No chill.

20th.—Temp. A.M., 97 2-5; 9 A.M., 97 4-5; P.M., 98. Bowels moved and headache gone. Feels "as well as ever in his life." Took the usual pill at at 11 A.M., 3 and 7 P.M.

21st.—Temp. A.M., 98. Duty. To take five grains of ipecacuanha, without opium, twice a day for some time.

23d.—Was seized with a chill while on guard last night (22d). Temp. A.M., 101 4-5; P.M, 99 4-5. To take ipecac. gr. x. (without opium) at 3 and 8:30 P.M. There was no chill, but he had a severe fever during the day.

24th.—Temp. A.M., 97 3-5; 3 P.M., 98 4-5; P.M., 100 2-5. ℞. Ipecac. gr. x. at at 6 and 11 A.M. and 5 and 8:30 P.M. There was no emesis, but a little nausea and anorexia.

25th.—Temp. A.M., 101 1-5; 1 P.M., 99 1-5; P.M., 98 2-5. Took ipecac. gr. v., opii gr. ¼ every four hours. Was nauseated all night, and had a chill, with high fever and perspiration, in the morning.

26th.—Temp. A.M., 98; P.M., 99 1-5. 27th, A.M., 98 2-5; P.M., 99 2-5. 28th, A.M., 98; P.M., 99. During each of these days he took four five-and-a-quarter-grain pills.

29th.—Temp. A.M., 99 2-5; P.M,, 98 3-5. Complaining of headache, the opium is omitted, and he is given three grains ipecacuanha every four hours.

30th.—Temp. A.M., 98 1-5; P.M., 101 1-5. Treatment continued, and ten grains ipecacuanha, without opium, given at night. No emesis.

31st.—Temp. A.M., 98; P.M., 99. Treatment continued, omitting the large dose.

1st February.—A.M., 97 3-5; P.M., 98 3-5. 2d, A.M., 97 4-5; P.M., 98 4-5. Treatment continued.

3d.—Duty, at his own request. To persevere with the three-grain pills.

12th February.—Reports another chill while on guard in very stormy weather. Taken on sick report and ordered ipecac. gr. ij. every two hours steadily. This treatment was not varied, and the temperature readings, night and morning, were as follows: 12th, 97 3-5, 99 4-5; 13th, 97 1-5, 100 1-5; 14th, 97 3-5, 99 2-5; 15th, 98, 100 1-5; 16th, 97 3-5, 90 2-5; 17th, 99 2-5, 99 2-5; 18th, 98 3-5. Duty. He had only one chill after he began to take the medicine, and he was retained on the sick list from the variations of temperature, although they were imperceptible to his sensations.

This man was again seized with a chill on the afternoon of 18th March, and had a very high fever that night. He reported sick the next day, and was given six one-grain pills of ipecacuanha, to be taken during the twenty-four hours. He had a lighter chill that evening, after which the disease ceased. The pills were continued four times daily for some days, and afterwards thrice and twice a day. He was returned to duty 24th March. I regret that the temperature was not taken during this attack, which was apparently as severe as any of the previous ones, and in the fever he was mildly delirious. It will be observed that doses of small size in this seemed more efficient than large doses in the earlier seizures. Up to the date of this paper he has remained in excellent health.

Case XII.—M. G. H. This man was one of a very few in his company who retained their health while on duty in Alabama in the fall of 1874.

19th January, 1875.—For the past three days he has felt chilly in the afternoon, with consecutive fever. Given by Asst. Surg. Maus ten 3-gr. quinine pills at 7 A.M., of which he took several (number not stated). Chilly sensations coming on in the evening, he was given, 6 P.M., in the hope of abating the paroxysm, chloroform. f℥ss., sp. ammon. arom. f℥j, aquæ f℥ij. This failed, and he was relieved from guard at midnight in consequence of the violence of the chill.

20th.—Admitted hospital, looking ill, with chilly sensations of body. Temp. A.M., 99 3-5; 5 P.M., 103; 7 P.M., 102 3-5. Given 10 A.M., ipecac. gr. xx., tr. opii *m.* xx. He remained in bed without nausea the most of the day, but on rising toward evening, to use the close-stool, vomited "a very little."

21st.—Temp. A.M., 97; 1 P.M., 102 1-5; 7 P.M., 103 1-5. Took ten grains ipecac. and half a grain opium at 10 A.M. and 6 P.M.

22d.—Temp A.M., 99 2-5; 1 P.M., 99 4-5; 3 P.M., 100 4-5; 7 P.M., 100 4-5. Took ipecac. gr. x., opii gr. ss. 6 A.M. and 1:30 P.M. The bowels not having moved for three days, given, 11 A.M., hydg. chl. mit. gr. x., pulv. ipecac., pulv. rhei aa. gr. v. One hour later the bowels moved and he vomited a little.

23d.—Temp. A.M., 98 3-5; P.M., 101 2-5. ℞. Ipecac. gr. x., opii gr. ss. 6 A.M., 1 and 8 P.M.

24th.—Temp. A.M., 98 3-5; P.M., 100 1-5. Took three grains every two hours.

25th.—Temp. A.M., 98; P.M., 99 2-5. Three grains every two hours.

26th.—Temp. A.M., 98; P.M., 99. Returned to quarters; treatment continued.

27th.—A.M., 97 3-5; P.M., 98 4-5. 28th, A.M., 97 4-5; P.M., 99 1-5. 29th, A.M., 98. 30th, A.M., 97 4-5; P.M., 99. 31st, A.M., 98; P.M., 98 3-5. 1st February, A.M., 98. 2d, 98, 99. 3d, A.M., 98 1-5. 4th, A.M., 98 1-5; P.M., 98 2-5. 5th, 98. Duty. There was no change in treatment since 25th January, and on going to duty he was supplied with pills to be taken for some days.

This man had no chill appreciable to himself after the second day he was on sick report, but he was kept from duty longer than usual because his countenance showed by its pallor the influence upon his system of the first violent seizure.

CASE XIII.—F. D. N. This man was relieved from guard in the night of 31st January, 1875, on account of a chill, and was given a dose of medicine, the memorandum of which is mislaid.

1st February.—Admitted hospital. Temp 8 A.M., 103 1-5; 1 P.M., 101 2-5; 4 P.M., 100 1-5; 6 P.M., 99 1-5. At 10 A.M., being constipated, was given hydg. chl. mit., gr. x., pulv. rhei, pulv. ipecac., aa gr. v. Bowels moved twice in the afternoon. At 8:30 P. M., given ipecac. gr. x. He was greatly nauseated, and vomited a little in the night.

2d.—Temp. A.M., 99; 1 P.M., 98 3-5; 6 P.M., 99 3-5. ℞. Ipecac. gr. v. at 10 A. M., and gr. ij. every two hours thereafter. No perceptible chill and no nausea. There was no indication of a chill after this date, and the temperature, under careful notings for five days, showed so little variation from the normal that it does not seem worth while to record it. The medicine was gradually diminished to two grains every four hours, which was kept up for some time.

The following case excellently illustrates the two hypotheses that pathological perspiration is indicative of disorder of the sympathetic, and that ipecacuanha is a direct corrective of the depression of that system.

CASE XIV.—W. M., a negro scavenger employed at the Post, complained, 29th January, 1875, of nightly pain proceeding from the pit of the stomach toward the chest, followed by cold sweats considerable in quantity and very annoying and debilitating. His health otherwise is good.

He was supplied with a quantity of two-grain pills of ipecacuanha, and was directed to take one every four hours.

3d February.—Night sweats have ceased, but he feels weak.

6th.—Has had no more perspiration and feels perfectly well.

Case XV.—This is a good example of the probable co-dependence of certain forms of intestinal disease and intermittents upon the same, or similar primary causes, as well as the control of both by the drug under discussion.

J. B. was on several occasions sick with some form of malarial fever while in Alabama in the autumn; but after his return to the post he remained well until the latter part of January, 1875, when the premonitory symptoms of ague came on, culminating in a chill, 3d February. A second and violent chill occurred before daylight on the 4th. Asst. Surg. Maus gave him hydg. chl. mit., jalap., aa gr. x.

5th.—A more severe chill, accompanied by excessive vomiting and followed by exhausting perspiration, occurred before daylight. Temp. 9 A.M., 96; 11 A.M., 97 1-5; 6 P.M., 97 2-5. Given two grains ipecac. every two hours and, the bowels moving too freely, a dose of Hope's mixture at 6 P.M.

6th.—Had a slight "crawling" sensation, but no chill this morning. Feels aguish and not well, but better. The bowels, however, have moved frequently with pain, and he has much painful irritation in the region of the transverse colon. Continued a two-grain pill every two hours, and at 11 A.M. gave ipecac. gr. xv., tr. opii *m.* x., in a paste with water. Became a little nauseated but did not vomit. The discharge from the bowels ceased at once, and the pain gradually disappeared. Temp. A.M., 96; P.M., 99 2-5.

7th.—Slept well and had no chill. The bowels moved naturally this morning without any pain. Temp. 9 A.M., 96 4-5; M., 97 3-5; 6 P.M., 98 1-5. Treatment continued.

8th.—Slept well, and had no discomfort of any kind. Temp. A.M., 97 1-5; P.M., 98 1-5. The treatment was kept up for some time, and there was no return of the disorder, nor did the temperature vary materially from the normal. He was retained on sick report until 15th, so that his strength, prostrated by the severity of the onset of the disease, might be regained.

Case XVI.—J. W. This man, who is reliable, reports, 12th March, 1875, that he had a marked chill last night, after one or two days' premonitory symptoms, and his countenance confirms his statement. Admitted hospital and given one grain of ipecacuanha every three hours. Temp. 8 P.M., 99 1-10; A.M., 98 4-5; M., 98 3-5; 2 P.M., 98 1-5; 4 P.M., 98 1-5; 7 P.M., 98.

13th.—Temp. 8 A.M., 97 1-5; 1 P.M., 98 3-5; 6 P.M., 98 3-5.

Treatment continued, and at 9 A.M. gave hydg. chl. mit., jalap., aa gr. x.; 3 P.M., mag. sulph. ℥ss.

14th.—Temp. A.M., 98; P.M., 98 3-5. One grain every four hours.

15th.—Medicine to be persevered with. Duty.

Although none occurred after this man's admission to hospital, I have no doubt that he had a severe chill, as he claimed. He suffered with acute enlargement of the spleen at the same time, which yielded to bromide of potassium, as advised by M. Bernard.

CASE XVII.—Lieutenant B. This officer, who complained that for some days he had badly-defined symptoms of malarial poisoning, which, about 17th March, 1875, developed into a tolerably evident chill, was directed to take one grain of ipecacuanha four times a day. There was no further chill, and upon his going to Chattanooga on the evening of 21st, he was supplied with a number, and told to take one three times a day. On his return, three weeks afterward, he reported his health as having been excellent.

CASE XVIII.—T. J. C. This man, a recruit, was admitted to hospital with a bronchial catarrh, 24th March, 1875.

1st April.—Says he had a chill about 3 P.M. Temperature 5 P.M., 103 1-5; 8 P.M., 102 1-5. No medicine.

2d—.Temp. 7 A.M., 97; 10 A.M., 97 3-5; 2 P.M., 98 2-5; 6 P.M., 99 1-5. No chill and no medicine.

3d.—Temp. 7 A.M., 98; 2 P.M., 98; 6 P.M., 98 2-5. No chill and no medicine.

4th.—Temp. 7 A.M., 98; 2 P.M., 98 1-5; 4 P.M., 101 4-5; 7 P.M., 103 1-5. Had a light chill about 3 P.M. ℞. Ipecac. gr. j every two hours.

5th.—Temp. A.M., 97; P.M., 98 4-5. No chill. Ipecac. gr. j every two hours.

6th.—Temp. A.M., 98 3-5; P.M., 98 1-5.

7th.—A.M., 95 3-5; 9 A.M., 99; 5 P.M., 100 4-5. A little feverish in the afternoon, but no chill.

8th.—7 A.M., 98 3-5; 6 P.M., 98 2-5.

9th.—7 A.M., 99 1-5. Returned to quarters, and ipecac. gr. j given every four hours. The treatment was continued, but the temperature was only taken on the evening of 13th, when he felt somewhat unwell, and it was found to be 98 3-5, and on the evening of 16th when, if at all, the quartan should reappear, at which time it was 98 2-5. 17th.—Duty.

CASE XIX.—J. F. H. An immature recruit, with a history of chills in Michigan, in September, 1874, and again for two days late in February, 1875, at Newport Barracks, Ky., claims to have had a chill on the night of 1st, and was admitted hospital for observation 2d April. Temp. 7 A.M., 97 3-5; 12:30 P.M., 98 1-5; 3 P.M., 98 3-5; 6 P.M., 98 4-5; 9 P.M., 100. No medicine.

3d.—Says he had a chill last night. Temp. 7 A.M., 98 4-5; 9 A.M., 98 4-5; 11 A.M., 99; 1 P.M., 99 4-5; 2 P.M., 97; 3 P.M., 101; 4 P.M., 103; 5 P.M., 105 1-5; 6 P.M., 104 3-5; 7 P.M., 104; 8:30 P.M., 102 1-5. Had a severe chill at 2 P.M. Bowels not having moved for several days, ℞. ol. tiglii. *m.* j at 5 P.M., and ipecac. gr. j every four hours. 9 P.M. vomited a little. Temp. 103. The chills that he had before taking the medicine prostrated him greatly and completely blanched his face.

4th.—Temp. 7 A.M., 98 3-5; 2 P.M., 98 2-5; 4 P.M., 99; 7 P.M., 99 1-5; 9 P.M., 101 3-5. Had some fever but no chill toward evening. Ipecac. continued. 3 P.M., ol. tiglii. *m.* ss. Bowels moved at night.

5th.—Temp. 7 A.M., 99 1-5; 1 P.M., 98 2-5; 3 P.M., 99 1-5; 6 P.M., 100.

6th.—Temp. 7 A.M., 98 4-5; 1 P.M., 98 1-5; 5 P.M., 98 1-5; 8 P.M., 98 1-5. No chill nor fever. Feels much better.

7th.—Temp. 7 A.M., 97; 3 P.M., 98 1-5; 7 P.M., 98 2-5. 8th, A.M., 98 2-5; P.M., 98 1-5. 9th, A.M., 97 3-5; P.M., 97 3-5. Feels very well but weak.

10th.—Temp. A.M., 96 4-5; P.M., 97 3-5. No chill, although the temperature is so low. To take for torpidity of bowels aloes, sapon., aa. gr. j., rhei. gr. ij, ipecac. gr. ¼. One or two at night.

11th.—Temp. A.M., 97; P.M., 97 2-5. 12th, A.M., 96 3-5; P.M., 98 1-5. 13th, A.M., 97 3-5; P.M., 98. 14th, A.M., 97 4-5; P.M., 98. 15th, A.M., 98 2-5; P.M., 98 3-5. Gaining strength slowly, was given tr. fer. chl. *m.* x., t.d. 16th, A.M., 98 3-5; P.M., 99.

17th.—Temp. A.M., 98 2-5. Sent to quarters. 6 P.M., returned to hospital, having just had a chill; temp. 98. 8:30 P.M., 101 1-5. To take one grain every three hours the next day. It will be noticed that the interval between these attacks was two weeks.

18th.—Temp. A.M., 98 1-5; 4:30 P.M., 99; 6 P.M., 99 3-5; 8:30 P.M., 100 1-5. A lighter chill.

19th.—Temp. 99 2-5, 98 2-5, 99 4-5; 20th, 97 1-5, 98 2-5, 98 3-5; 21st, 98 3-5, 98 2-5. Claims to have had light chills on the last three days.

22d.—Temp. 7 A.M., 97 2-5; 9 A.M., 97 2-5; 11 A.M., 97 2-5; 1 P.M., 97 3-5; 3 P.M., 98 1-5; 5 P.M., 98 2-5; 7 P.M., 99 2-5.

1st May.—Duty.

There has been no recognizable chill since 18th, and he is regaining strength slowly.

Case XX.—Mrs. ......, an officer's wife. This lady, who has spent a number of years in this latitude, has every spring an ill-defined malarial attack, generally culminating in an ague. About the middle of March she complained of impaired appetite, languor, fugitive pains and headache, developing into a slight but marked chill. She also had occasional feelings of weight and pressure in the abdomen, and some spinal tenderness was found. Quinine, which she had been accustomed to use, has very disagreeable effects. I therefore gave her a one-grain pill of ipecacuanha four times a day. She also was given an ammoniacal liniment, as a spinal counter-irritant. The first dose nauseated her, but she did not vomit. She had no further annoyance from the medicine until after the entire disappearance of the morbid symptoms, which occurred soon. The nausea then again set in, and the ipecacuanha was suspended.

In this case the chills ceased at once, the pains subsided, the spinal tenderness disappeared, and the appetite, digestion and spirits became natural within a very few days.

Case XXI.—D., an elderly colored woman, complained of almost identical symptoms with Case XX., excepting the spinal tenderness. She was given one grain four times a day, and in a few days was completely restored.

Case XXII.—D. H. W., a recruit, admitted hospital 4 p.m., 14th April, 1875, having just had a heavy chill. Temp. 4 p.m., 101 3-5; 6 p.m., 102 3-5. ℞. Ipecac. gr. j every three hours.

15th.—Temp. 98; 2 p.m., 98; 6 p.m., 99 1-5. No chill but much aching in the legs. Treatment continued. 16th, a.m., 98 1-5; p.m., 98 3-5. As yesterday but better. 17th, a.m., 96 1-5; 3 p.m., 97; 6 p.m., 97 3.5. No chill. 18th, a.m., 95 1-5; 10 a.m., 96 3-5. Notwithstanding this extremely low temperature, which was carefully taken, he had no unnatural feelings except the general aching.

20th.—Duty, with directions to continue the treatment, which had been adhered to from the first.

Case XXIII.—Captain K. This officer, who has suffered much from malarial infection, is subject to violent cranial neuralgia, for which quinine, which he habitually keeps in his house and takes at discretion, has been the usual and most satisfactory remedy. Having had severe and almost constant neuralgia for several days, he took nine grains of quinine night and morning for two successive days without producing any impression. He applied for treatment 19th April, when I suspended the quinine and supplied him with one-grain pills of ipecacuanha, one to be taken every four hours. After the fifth pill the pain ceased. He then increased the intervals and the pain recurred the following night ( 3 A.M. 21st,) but stopped after the third pill. On the day after (22d,) maxillary neuralgia set in, but ceased after the second dose. He continued the medicine until nausea occurred that night, after which it was taken only twice a day. He has remained well to this date.

I do not care at this time to discuss the pathological or therapeutical points involved, nor to refer to the literature of the subject, desiring merely to present certain clinical experience as a contribution to the facts of medicine, and thus to incite others to similar observations and reports.

The series from which the foregoing are abstracted represents twenty-eight individuals and, including the recurrences, embraces thirty-seven cases of disease unselected and consecutive, being all of that class that have come under my observation during the past five months. The following is a summary of them.

In one, not entered here, aguish symptoms during eight days appeared to be held in check by ipecacuanha, but pneumonia followed which was recovered from under ordinary treatment.

Case IV., anomalous and troublesome, has been analysed.

Cases XIV, of pathological perspiration, and XXIII, of severe neuralgia, both of which might be regarded as masked intermittent, responded at once to the medicine.

Of the remaining twenty-four individuals, one tertian (Case II) relapsed in four weeks, but no chill occurred after the renewal of the medicine; one quotidian (Case V) had a recurrence in two days, following drunkenness, when there were two chills after resuming treatment, and again in two weeks when they ceased at once; one tertian (Case VII) relapsed after two months, but had no chill after resuming treatment; one quotidian (Case VIII) recurrred in two weeks, but stopped with one light chill; one severe quotidian (Case XI) relapsed in two days and again in two weeks during exposure to storms, and again in four weeks, on only two of which occasions a single chill was manifest; and one quotidian, not reported, had one chill after two weeks' suspension. Thus it is seen that of the nine recurring cases, (II., V., VII., VIII., XI., XIX.—seven men,) in only one case, (V.) did two chills persist, in four cases, (VIII., XI., XIX.—three men,) one chill each, and in four (II., V., VI., XI.,) no chill was felt after the medicine was resumed.

Of the twenty-four original intermittents, one only, (Case VII.) had two light chills, ten (II., III., V., VI., XI., XVIII., the others not reported here,) had but one each, and the remaining thirteen (I., VIII., IX., X., XIII., XV., XVI., XVII., XIX., XX., XXI., XXII., the other not reported here,) had no chill after beginning with the ipecacuanha, a result that, in my opinion, at least equals what might be looked for from quinine.

Two cases (V., XV.,) illustrate the occasional co-existence of dysenteric symptoms with intermittents and the prompt suppression of the former by large doses of ipecacuanha.

I repeat the remark that I believe all the cases reported are genuine, and the men experienced exactly the symptoms described. In taking the temperature, the bulb of the instrument was habitually placed under the tongue. The men were kept much longer on the sick list than is usual, on account of the fluctuations of temperature shown by the thermometer, although these were not recognized by the patients. In ordinary practice, such cases are styled cured as soon as the chills cease, and I do not think that the circumstance that these men were detained from duty for a longer time should militate against the therapeutical record. In like manner the fact that no adjuvants were employed in some instances, when quite clearly indicated, should be borne in mind in computing the influence of the drug.

The very trivial emesis that occurred once or twice and the occasional nausea do not, I think, forbid the epithet 'non-nauseating' as applied to the treatment, although I believe a good preliminary emetic would sometimes be useful.

In this series the medicine was given in varying quantities, from one grain to twenty. My present impression is, that one or two grains every three or four hours is the best method for ordinary chills. Large doses did not appear to exert a beneficial effect in proportion to their size. If large doses are used, it is well to guard them with small quantities of opium and to observe the precautions of recumbent rest and abstinence. In one-grain doses, abstinence from fluid is usually all that is necessary. I think that I have observed that the stomach tolerates the drug in proportion to its needs.

Taking the facts herein reported in connection with those in a previous paper by the writer, and with certain hypotheses there advanced, the possible usefulness of ipecacuanha in some forms of the malarial hæmaturia of the South is suggested.

The method herein illustrated seems to offer substantial and obvious advantages, but it will require numerous and careful experiments to determine its exact value. Incomplete or partial testimony will be worse than useless.

Careful independent thermometrical and other observations upon intermittents, especially under different modes of treatment, are very desirable, and these may easily be carried on in public institutions. If the Medical Corps of the Army would undertake such an investigation *con amore*, the position of ipecacuanha as an antiperiodic might be settled within a year.

McPherson Barracks,
*Atlanta, Ga., May* 1, 1875.

